# Updates in the management of unknown primary of the head and neck

**DOI:** 10.3389/fonc.2022.991838

**Published:** 2022-09-15

**Authors:** Sandhya Kalavacherla, Parag Sanghvi, Grace Y. Lin, Theresa Guo

**Affiliations:** ^1^ School of Medicine, University of California, San Diego, La Jolla, CA, United States; ^2^ Department of Radiation Medicine and Applied Sciences, University of California, San Diego, San Diego, CA, United States; ^3^ Department of Pathology, University of California, San Diego, San Diego, CA, United States; ^4^ Department of Otolaryngology – Head & Neck Surgery, University of California, San Diego, San Diego, CA, United States

**Keywords:** human papillomavirus - HPV, oropharyngeal squamous cell carcinoma (OPSCC), unknown primary head and neck squamous cell carcinoma, head and neck cancer, management

## Abstract

Squamous cell carcinoma (SCC) from an unknown primary tumor (SCCUP) accounts for 2.0%–5.0% of all head and neck cancers. SCCUP presents as enlarged cervical lymph nodes without evidence of a primary tumor upon physical examination. Primary site detection is important to target treatment and avoid treatment-related morbidity. In this review, we discuss updates in SCCUP management. Diagnostic workup should focus on localization of the primary tumor in SCCUP. Initial workup centers on neck biopsy to confirm the presence of SCC. Given the increasing incidence of HPV-related SCC in the oropharynx, HPV testing is crucial. An HPV-positive status can localize the tumor to the oropharynx, a common site for occult tumors. Imaging includes neck CT and/or MRI, and PET/CT. After imaging, panendoscopy, palatine tonsillectomy or diagnostic transoral robotic surgery can facilitate high rates of primary tumor localization. Primary tumor localization influences treatments administered. SCCUP has traditionally been treated aggressively with large treatment fields to all potential disease sites, which can induce weight loss and swallowing dysfunction. As a result, primary localization can reduce radiation fields and provide possible de-escalation to primary surgical management. Advances in intensity-modulated radiation therapy and dose management also have the potential to improve functional outcomes in SCCUP patients. Given the improved prognosis associated with HPV-positive SCCs, HPV tumor status may also inform future treatment de-intensification to reduce treatment-related toxicity.

## Introduction

The global incidence of head and neck squamous cell carcinoma (HNSCC) is anticipated to increase by 30% to 1.08 million new cases annually by 2030 (1). HNSCC from an unknown primary tumor (SCCUP) accounts for 2.0%–5.0% of all head and neck cancers (2), presenting a significant diagnostic and therapeutic challenge. SCCUP is defined as metastatic squamous cell carcinoma (SCC) to cervical lymph nodes without evidence of a primary tumor upon physical examination. A primary tumor can evade detection due to a combination of its location, small size, and potential regression of the primary (3).

Recently, SCCUP incidence has increased significantly, primarily driven by HPV infection (4). In the United States, rates of tobacco related, HPV-negative HNSCCs are decreasing (5), given decreasing tobacco consumption since the 1960s (6). Simultaneously, oropharyngeal HPV infection rates have significantly increased in the last 20 years (5). The incidence of HPV-positive HNSCCs in the United States increased by approximately 225% from 1988 to 2004, while incidence for HPV-negative HNSCCs decreased by 50% (7). In fact, HPV-associated oropharyngeal SCCs (HPV-OPSCC) have surpassed cervical cancers as the most common HPV-related cancer (8). HPV-OPSCC may present as occult primary tumors in the crypt epithelium of the palatine or lingual tonsils (9), thus evading surface detection and presenting as SCCUP.

The ideal SCCUP treatment remains controversial, given the paucity of randomized controlled trials informing treatment targets. Consequently, extensive diagnostic workup is essential to localize the primary site. However, despite exhaustive efforts to find the primary site, overall rates of primary detection are suboptimal, reported as low as approximately 50% (3, 10). Since treatment of HNSCC is largely informed by the primary site, SCCUP patients pose a unique challenge. In this review, we discuss updates in the diagnostic workup and treatment of SCCUP.

## Physical exam and clinical history

A SCCUP patient usually presents to the clinician with cervical lymphadenopathy, appearing as a persistent, painless, and mobile neck mass in levels II-III. Other etiologies of a neck mass are considered, including infection, inflammation, congenital lesions, or other neoplasms, such as lymphomas (11). Symptoms of dysphagia, odynophagia, otalgia or weight loss increase initial suspicion for mucosal origin, and additional aspects of a history such as gender, age, tobacco use, sexual history, and history of cutaneous or other solid malignancies can give evidence towards primary diagnosis (2).

A primary tumor is often difficult to detect on physical examination, but small primaries can sometimes be identified using distal chip flexible laryngoscopy. Flexible endoscopy with narrow band imaging (NBI) is a new technology that highlights neo-angiogenesis to provide superior visualization of mucosa compared to standard endoscopy (2, 12). Studies report successful primary detection using NBI in SCCUP cases where traditional workup did not localize a primary site, with a pooled detection rate of 35%, sensitivity of 83%, and specificity of 88%. (2, 13) Ebisumoto et al. (14) specifically demonstrate increased detection of HPV-related oropharyngeal primary tumors when using transoral NBI endoscopy, highlighting its non-invasiveness and feasibility in outpatient settings.

## Neck biopsy

Biopsy of the neck mass ascertains the presence of SCC over other etiologies. Fine needle aspiration (FNA) is the first-line tool as it is minimally invasive and cost-effective (15). FNA should be ultrasound-guided, to ensure accuracy of tissue sampling and reduce non-diagnostic samples (16). In particular, HPV-positive SCC often presents with cystic nodes, and biopsy should be targeted toward the periphery to ensure adequate cellularity for diagnosis. FNA has high specificity and sensitivity. A meta-analysis reports that FNA of cervical lymph nodes had a sensitivity of 94.2% and specificity of 96.9%, while FNA of the major salivary gland, thyroid gland, and other sites, including cystic neck masses and oral cavity lesions, had sensitivities and specificities of 85.5% and 98.4%; 79.7% and 98.1%; 78.7% and 97%, respectively (15).

Core needle biopsy (CNB) uses a cutting needle piston, which obtains a larger tissue sample to preserve the native histologic architecture (17). One meta-analysis comparing FNA and CNB reports that CNB can achieve a higher accuracy in detecting malignancy (17). Another study reports accuracy, sensitivity, and specificity values of CNB as 94%, 92% and 100%, respectively (18). CNB is also useful in additional histopathological analysis, such as determining p16 or HPV status (2).

In up to 10% to 15% of cases, FNA may be insufficient in supplying enough diagnostic material (2). Excisional biopsy, a procedure in which the entire mass is removed and examined, should only be reserved for cases where needle biopsy cannot provide a reliable diagnosis (17). Some studies suggest that excisional biopsies result in a “violated neck” which may be associated with wound compilations and higher recurrence (19), although this has not been uniformly reported (20, 21). If proceeding with excisional biopsy, the surgeon must be prepared to perform a complete neck dissection if pathology demonstrates carcinoma (2).

HPV testing of nodal tissue is critical in SCCUP workup because HPV positivity localizes the primary tumor to the oropharynx. HPV status is determined by immunohistochemical (IHC) detection of p16^INK4a^, a marker for HPV E7 oncogene expression (2). Among patients who underwent FNA, one study reports that p16 positivity in nodal sites was predictive of oropharyngeal origin and had a 98% correlation with HPV *via* HPV DNA *in situ* hybridization (ISH) (22). Current guidelines recommend optional confirmatory testing through HPV DNA ISH or PCR if p16 IHC yields ≥70% staining of tumor cells (2). A limitation of p16 testing for HNSCC is that elevated p16 can also be present in non-HPV disease outside the oropharynx, such as lymph node-positive cutaneous SCCs. One study found that approximately 6% of metastatic SCCs in the neck were p16-positive and HPV-negative with confirmed primary sites outside of the oropharynx (23). However, there is limited data on p16 elevation rate in cutaneous primaries (24). Since using p16 expression as the sole biomarker to localize an unknown primary to the oropharynx is not always reliable, the possibility of a cutaneous primary should be ruled out (24). High tumor mutational burden or UV mutation signatures can be utilized to identify a cutaneous primary (25).

HPV negative tumors can be further tested for EBV using ISH, which can localize the tumor to the nasopharynx. One retrospective study showed that among patients with EBV-positive nodes, 51.7% of the primary sites were in the nasopharynx (26).

## Imaging

Imaging is essential to identifying a primary tumor, and suspicious sites on imaging are biopsied. Due to its availability and low cost, contrast-enhanced computed tomography scan (CT) of the neck with contrast is commonly the first-line imaging tool (2). Magnetic resonance imaging (MRI) is also increasingly used, as MRI can provide higher resolution, better delineation of tumor margins, and superior detection of small oropharyngeal tumors in patients with p16 positive lymph nodes (2). Detection of the primary site using CT and/or MRI in patients with no suggestive findings on physical examinations has been reported between 33% and 50% (27, 28). A meta-analysis of studies comparing CT and MRI found that CT had a higher sensitivity (77% vs 72%) but lower specificity (72% vs 81%) compared to MRI (2).

18F-fluorodeoxyglucose-positron emission tomography (PET) scans are another key imaging modality for identifying primary sites in SCCUP patients. A study comparing the diagnostic accuracy of PET alone with integrated PET and CT (PET/CT) demonstrated that PET/CT had a significantly higher primary detection and positive prediction rate compared to PET alone (29). Primary detection in SCCUP patients *via* PET/CT has been reported as ranging from 17% to 55.2% (29, 30). Other studies report PET/CT sensitivity ranging from 79.2% to 91.5% and specificity ranging from 70.4% to 87% (2). PET/CT is limited in detecting primary tumors less than 10 mm and those in the crypts of the lingual tonsillar tissue of the base of tongue (2). In addition, the oropharynx often demonstrates physiologic FDG avidity that may obscure small tumors (31).

## Panendoscopy and tonsillectomy

To pathologically confirm the primary site, panendoscopy and/or tonsillectomy can be performed. Panendoscopy includes direct laryngoscopy, bronchoscopy, and esophagoscopy performed under general anesthesia, allowing for inspection of at-risk mucosa (2). Only sites suspicious for cancer, such as those with irregularities in the mucosa and abnormal bleeding, are biopsied, as random biopsies are considered low yield (32).

An advantage of panendoscopy is its ability to detect synchronous primary tumors, which can occur with chronic tobacco and alcohol exposure but are rare in patients with HPV-positive disease (33). Given the decreasing incidence of tobacco-associated HNSCCs coupled with the rising incidence of HPV-positive HNSCC and introduction of PET/CT, the utility of panendoscopy for SCCUP patients has been questioned. While studies report a primary detection rate of approximately 10% *via* panendoscopy in patients with negative imaging, some argue that this benefit to only 10% of SCCUP patients must be considered against the disadvantages of panendoscopy, including the cost and risks of general anesthesia (34). Other studies support the selective use of panendoscopy. Noor et al. (33) suggest that panendoscopy can assess suitability for transoral robotic surgery (TORS) and identify synchronous tumors in high-risk patient groups. Similarly, Metzger et al. (35) support risk stratification before panendoscopy use in order to reduce unnecessary procedures.

In cases with negative directed biopsies from panendoscopy, ipsilateral palatine tonsillectomy can be performed, which has a reported additional primary detection rate of up to 50% (2). For patients with bilateral lymphadenopathy, palatine tonsillectomy is recommended first on the side with the greater nodal burden (2). If this procedure cannot identify the primary, contralateral palatine tonsillectomy can be considered (2). A main advantage of tonsillectomy is its feasibility in the community setting and decreased invasiveness compared to TORS, although tonsillectomy still holds potential risk for post-operative hemorrhage.

## Diagnostic transoral robotic surgery (TORS)

**Figure 1:**
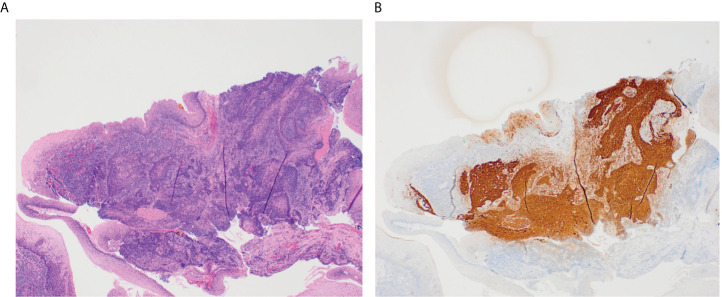
These slides demonstrate a small 3 mm tumor in the glossotonsillar sulcus that was identified through TORS. The H&E stained image of this tumor shows irregular nests of non-keratinizing squamous cell carcinoma underlying normal squamous mucosa in a background of tonsillar lymphoid tissue **(A)**. P16 immunostain is diffusely positive in ~100% of tumor cells **(B)**.

When above efforts fail to identify a primary tumor, patients can undergo TORS, which improves visualization of the oropharynx and facilitates lingual tonsillectomy or ipsilateral oropharyngectomy to identify otherwise occult primaries (2, 36). TORS has success in identifying hidden oropharyngeal tumors (**Figure 1**). Hatten et al. (37) report that TORS facilitated the identification of 80% of occult oropharyngeal tumors. Other studies report primary site identification rates *via* TORS ranging from 72% to 94% (2, 37, 38).

Another benefit to using TORS is the possibility to accomplish diagnosis and resection of tumor in the same session, which occurred in 76.5% of diagnostic TORS cases in one study (36). TORS is an invasive procedure and can induce adverse effects, including dysphagia, bleeding, airway edema, and death (39). Bleeding rates from TORS have been reported as ranging from 0.5% to 10.4% (39); however, diagnostic TORS has lower bleeding rates than oncologic TORS (2). Patel et al. (40) found better preserved swallowing function among SCCUP patients who underwent diagnostic TORS compared to patients who underwent TORS-mediated resection of clinically identified tumors. External carotid branch ligation is also now routinely performed to reduce the risk of life-threatening bleeding during TORS (41, 42).

The use of diagnostic transoral robotic oropharyngectomy is highest yield in work up of HPV-positive SCCUP, with HPV-positive tumors comprising 55-96% of all tumors found by this method (43). HPV-negative patients, however, may be less likely to benefit from TORS with detection rates as low as 13%, and the risks may not outweigh benefits (44).

## Treatment based on primary localization

HNSCC of known primary may be treated with resection of the primary tumor and adjuvant therapy, if necessary. Conversely, SCCUP is often treated with large radiotherapy fields, despite the evidence that such aggressive treatment causes adverse outcomes.

## De-escalation based on primary identification

Primary tumor identification *via* TORS facilitates treatment de-escalation. Durmus et al. (36) report that the detection and primary tumor resection with TORS both focused the adjuvant treatment regimen and also de-intensified it by decreasing the radiotherapy dose to the entire upper aerodigestive tract and avoiding chemotherapy. Similarly, among their cohort of patients with tumors found *via* TORS, Hatten et al. report that the overwhelming majority of these patients were diagnosed with stage IV HNSCC but did not receive chemotherapy despite national guidelines. Instead, they were treated with TORS-mediated tumor resection and neck dissection. The authors cite the high rate of esophageal strictures and swallowing deficits from the traditional chemotherapy regimen for stage IV HNSCC as the rationale to de-escalate treatment to surgery. Patel et al. (45) similarly report that TORS-workup of SCCUP facilitated primary identification in 74.3% of patients, resulting in de-escalation to surgical management and dose and volume reduction of adjuvant radiation. Specifically, among the 26 patients with primaries found *via* TORS, 46.1% had lower radiation volumes, and 30.1% had the contralateral neck spared from radiation.

## Radiation fields

In the era before widespread HPV testing and exhaustive diagnostic workup tools, SCCUP was treated aggressively with radiation to the bilateral neck and mucosa in the entire pharyngeal axis, including the nasopharynx, oropharynx, larynx, and hypopharynx (46). However, routine radiation to all possible primary sites did not necessarily improve survival (47). Historically, patients with multi-nodal involvement and no smoking history received mucosal radiation to the nasopharynx, oropharynx, and the bilateral neck at 50 Gy, and the gross disease was treated at 70 Gy. If the patient had a smoking history, the entire pharyngeal axis was treated at 50 Gy, which often led to swallowing dysfunction.

In the modern era, efforts are being made to spare the pharyngeal axis *via* extensive diagnostic workup. EBV and HPV status can focus treatment, as EBV-positive disease directs treatment to the nasopharynx and HPV-positive disease limits treatment to the oropharynx, which has yielded acceptable outcomes that do not compromise survival or local tumor control (48). If all primary localization efforts are unsuccessful and the SCCUP patient has multi-nodal involvement, the patient is treated with a non-surgical pathway involving radiotherapy similar in principle to that from the era before HPV testing. Notably, the majority of SCCUP diagnosed today are HPV-positive, resulting in few patients requiring radiation to the entire pharyngeal axis (4).

Given the morbidity of large volume mucosal irradiation, sophisticated treatment planning techniques using either intensity modulated radiotherapy (IMRT) or protons are preferred (49, 50). IMRT avoids healthy tissue exposure and has a lower toxicity profile (51). While high locoregional tumor control has been reported with IMRT use in SCCUP patients, advances are still needed in toxicity reduction and managing patients prone to distant metastases (52, 53). Further, among SCCUP patients treated with IMRT, studies report rates of high-grade xerostomia ranging from 5-36% at 6 months and 0-15% at 24 months after treatment, and rates of feeding tube dependence ranging from 0-5% at 12 months after treatment (53).

Grewal et al. (54) compared the effects of pharyngeal-sparing radiotherapy (PSRT) to pharyngeal-targeted radiotherapy (PRT) in the post-TORS adjuvant setting for SCCUP treatment and report reduced toxicity following PSRT. In their study, PSRT was associated with statistically significantly lower mean weight loss, feeding tube placement, new opioid requirement, and unplanned hospitalizations during radiation treatment compared to PRT. With identification and resection of the primary tumor, PSRT may be considered as a de-escalation strategy.

## HPV tumor status

HPV status has important prognostic significance, which influences the appropriate SCCUP treatment. It is well-known that HPV-positivity is a strong, positive prognostic factor for oropharyngeal SCCs (55). Possible confounders of the improved prognosis in HPV-positive disease include the younger ages and lower comorbidity indexes among HPV-positive patients compared to HPV-negative patients (5). As previously discussed, an HPV-positive status allows for oropharynx-focused radiation fields, which spares the larynx and reduces of the risk of voice loss, swallowing dysfunction, and feeding tube reliance (54, 56, 57).

Other studies have investigated the prognosis of HPV-positive SCCs in areas outside of the oropharynx (non-OPSCC). Ko et al. (58) suggest that patients with HPV-positive non-OPSCC had similar characteristics as patients with HPV-OPSCC. Other studies similarly support favorable prognosis of HPV-positive non-OPSCC (59–62), while some report the contrary (63). While HPV-positive SCCUP is generally presumed to be of oropharyngeal origin, these improved prognoses may be translatable to HPV-positive patients with persisting unknown primaries.

## Discussion

Major advances have been made in the past two decades to improve SCCUP treatment, including TORS development to increase primary detection and IMRT adoption to reduce treatment morbidity. Future challenges to improving SCCUP outcomes include increasing specialized care access, improving long-term functional outcomes, and incorporating HPV tumor status into treatment de-escalation when appropriate.

**Figure 2:**
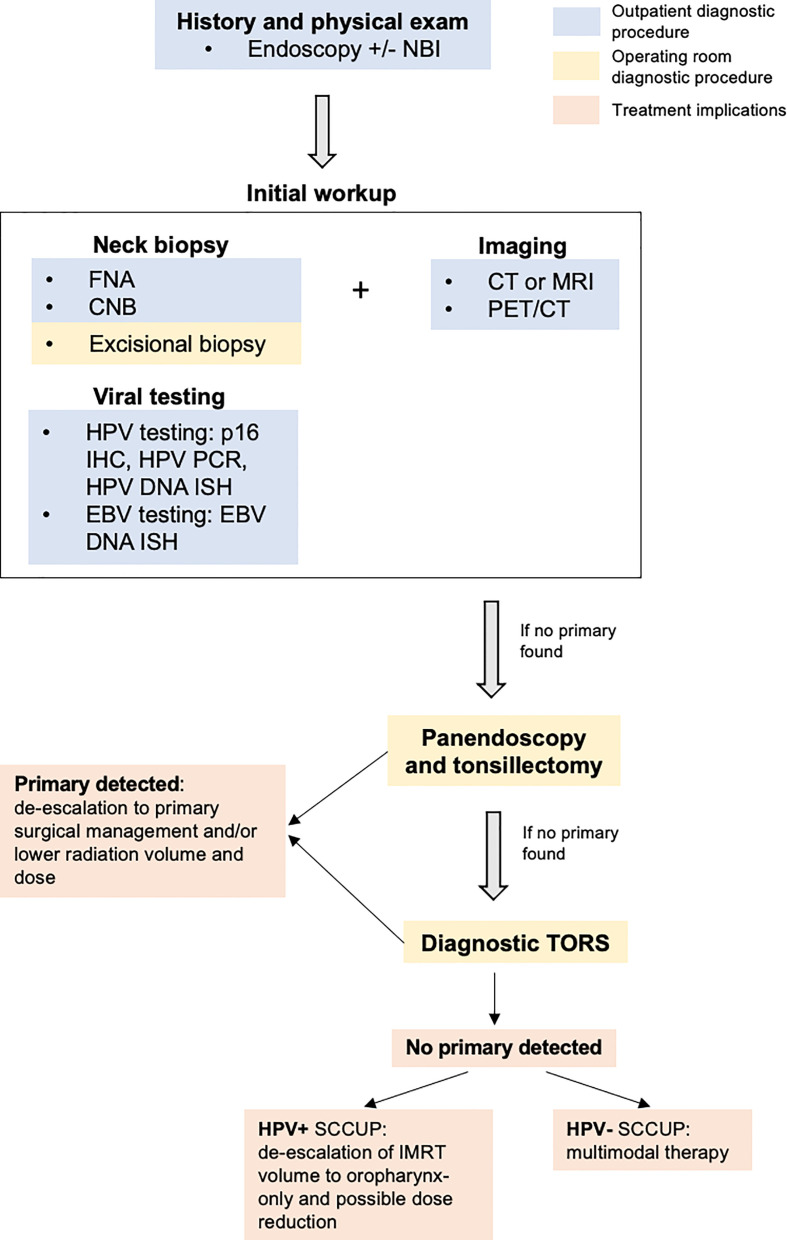
Overall diagnostic workup and treatment implications for a patient who presents to clinic with a neck mass.

Primary tumor detection plays a critical role in a treatment regimen and subsequent outcomes, and a full diagnostic workup is outlined in **Figure 2**. While TORS has a reported detection rate as high as 94% (38), unknown primary detection rates are as low as approximately 50% in clinical practice (3, 10). TORS is not universally available at all facilities due to need for specialized equipment and training. An NCDB analysis demonstrated that SCCUP patients treated at community practices had significantly worse outcomes with decreased overall survival (64). While the exact etiology of the poorer outcomes is unknown, few non-academic centers offer TORS and subsequent radiation may not be administered by providers with specific head and neck experience. Imaging advances may reduce dependence on TORS for primary tumor identification in low-resourced settings. However, a future challenge is to promote widespread TORS access and tertiary center referral for SCCUP treatment.

Improvements in long-term swallowing and functional outcomes for SCCUP patients are still needed. While IMRT is adopted as the primary radiation therapy for SCCUP, improvements to its administration can reduce toxicity (65). LaVigne et al. (57) investigated mucosal dose-related effects of IMRT in SCCUP patients, finding that a 56 Gy IMRT-based mucosal dose and larynx-sparing IMRT were associated with reduced swallowing toxicity. However, more research on dose-related IMRT toxicity is required in this field to elucidate ideal doses for SCCUP patients with varying levels of nodal involvement and the interaction between IMRT dose and adjuvant chemotherapy. Additionally, different practices in choosing radiation fields must be considered. While Grewal et al. showed PSRT post-TORS resection could improve functional outcomes, this practice is not widely adopted as the standard of care for SCCUP.

Given evidence supporting the favorable prognosis in HPV-positive HNSCC, an HPV-positive status has the potential to inform treatment deintensification among SCCUP patients. While current guidelines do not yet specifically discuss the use of an HPV status to de-escalate treatment, several de-escalation trials for HPV-related disease have recently been published or are underway (66, 67). Data is also limited on appropriate treatment for HPV-negative SCCUP. Cheraghlou et al. (68) demonstrate significant differences in survival based on treatment modality among HPV-negative SCCUP patients. They report that the use of multiple modality therapy, either chemoradiotherapy or surgery with adjuvant chemoradiotherapy, resulted in improved survival compared to use of radiotherapy alone. However, multiple modality therapy increases risk for treatment-related morbidity. Further, for early-stage HPV-negative oropharyngeal SCC, surgery may offer improved outcomes over chemoradiation, given reduced efficacy of non-surgical therapies (69). Similar concepts may be translatable to HPV-negative SCCUP and such trials investigating treatment options for HPV-negative are needed.

## Author contributions

Study conception and design, writing: TG, SK, Literature review, analysis, writing: SK and PS, Pathology images, writing: GL. All authors contributed to the article and approved the submitted version.

## Funding

TG is supported by 1KL2TR001444 through NIH.

## Conflict of interest

The authors declare that the research was conducted in the absence of any commercial or financial relationships that could be construed as a potential conflict of interest.

## Publisher’s note

All claims expressed in this article are solely those of the authors and do not necessarily represent those of their affiliated organizations, or those of the publisher, the editors and the reviewers. Any product that may be evaluated in this article, or claim that may be made by its manufacturer, is not guaranteed or endorsed by the publisher.

## References

[1] Johnson DE Burtness B Leemans CR Lui VWY Bauman JE Grandis JR. Head and neck squamous cell carcinoma. Nat Rev Dis Primers (2020) 6(1):92. doi: 10.1038/s41572-020-00224-3 33243986 PMC7944998

[2] Ye W Arnaud EH Langerman A Mannion K Topf MC. Diagnostic approaches to carcinoma of unknown primary of the head and neck. Eur J Cancer Care (Engl) (2021) 30(6):e13459. doi: 10.1111/ecc.13459 33932056

[3] Lee MY Fowler N Adelstein D Koyfman S Prendes B Burkey BB. Detection and oncologic outcomes of head and neck squamous cell carcinoma of unknown primary origin. Anticancer Res (2020) 40(8):4207–14. doi: 10.21873/anticanres.14421 32727746

[4] Motz K Qualliotine JR Rettig E Richmon JD Eisele DW Fakhry C. Changes in unknown primary squamous cell carcinoma of the head and neck at initial presentation in the era of human papillomavirus. JAMA Otolaryngol Head Neck Surg (2016) 142(3):223–8. doi: 10.1001/jamaoto.2015.3228 26769661

[5] Rettig EM D’Souza G. Epidemiology of head and neck cancer. Surg Oncol Clin N Am (2015) 24(3):379–96. doi: 10.1016/j.soc.2015.03.001 25979389

[6] Rodu B Cole P. Declining mortality from smoking in the united states. Nicotine Tob Res (2007) 9(7):781–4. doi: 10.1080/14622200701397957 17577808

[7] Chaturvedi AK Engels EA Pfeiffer RM Hernandez HY Xiao W Kim E. Human papillomavirus and rising oropharyngeal cancer incidence in the united states. J Clin Oncol (2011) 29(32):4294–301. doi: 10.1200/JCO.2011.36.4596 PMC3221528 21969503

[8] Lechner M Liu J Masterson L Fenton TR. HPV-associated oropharyngeal cancer: epidemiology, molecular biology and clinical management. Nat Rev Clin Oncol (2022) 19(5):306–27. doi: 10.1038/s41571-022-00603-7 PMC8805140 35105976

[9] Channir HI Grønhøj Larsen C Ahlborn LB Hansen TO Gerds TA Charabi BW. Validation study of HPV DNA detection from stained FNA smears by polymerase chain reaction: Improving the diagnostic workup of patients with a tumor on the neck. Cancer Cytopathol (2016) 124(11):820–7. doi: 10.1002/cncy.21753 27404322

[10] Ryan JF Motz KM Rooper LM Mydlarz WK Quon H Gourin CG. The impact of a stepwise approach to primary tumor detection in squamous cell carcinoma of the neck with unknown primary. Laryngoscope (2019) 129(7):1610–6. doi: 10.1002/lary.27625 30565698

[11] Schwetschenau E Kelley DJ. The adult neck mass. Am Fam Phys (2002) 66(5):831–8. 12322776

[12] Di Maio P Iocca O De Virgilio A Giudice M Pellini R D’Ascanio. Narrow band imaging in head and neck unknown primary carcinoma: A systematic review and meta-analysis. Laryngoscope (2020) 130(7):1692–700. doi: 10.1002/lary.28350 31714611

[13] Shinozaki T Hayashi R Ebihara M Miyazaki M Daiko H Saikawa M. Narrow band imaging endoscopy for unknown primary tumor sites of the neck. Head Neck (2012) 34(6):826–9. doi: 10.1002/hed.21825 21853500

[14] Ebisumoto K Sakai A Maki D Robinson K Murakami T Iijima H. Tumor detection with transoral use of flexible endoscopy for unknown primary head and neck cancer. Laryngosc Investig Otolaryngol (2021) 6(5):1037–43. doi: 10.1002/lio2.656 PMC8513428 34667847

[15] Tandon S Shahab R Benton JI Ghosh SK Sheard J Jones TM. Fine-needle aspiration cytology in a regional head and neck cancer center: comparison with a systematic review and meta-analysis. Head Neck (2008) 30(9):1246–52. doi: 10.1002/hed.20849 18528906

[16] Baatenburg de Jong RJ Rongen RJ Verwoerd CD van Overhagen H Lameris JS Knegt P. Ultrasound-guided fine-needle aspiration biopsy of neck nodes. Arch Otolaryngol Head Neck Surg (1991) 117(4):402–4. doi: 10.1001/archotol.1991.01870160056008 2007009

[17] Novoa E Gürtler N Arnoux A Kraft M. Role of ultrasound-guided core-needle biopsy in the assessment of head and neck lesions: a meta-analysis and systematic review of the literature. Head Neck (2012) 34(10):1497–503. doi: 10.1002/hed.21821 22127851

[18] Ferreira VHC Sassi LM Zanicotti RTS Ramos GHA Jung JE Schussel JL. Core needle biopsy in the diagnosis of head and neck lesions: a retrospective study of 3 years. Eur Arch Otorhinolaryngol (2016) 273(12):4469–72. doi: 10.1007/s00405-016-4139-6 27295173

[19] McGuirt WF McCabe BF. Significance of node biopsy before definitive treatment of cervical metastatic carcinoma. Laryngoscope (1978) 88(4):594–7. doi: 10.1002/lary.1978.88.4.594 642657

[20] Razack MS Sako K Marchetta FC. Influence of initial neck node biopsy on the incidence of recurrence in the neck and survival in patients who subsequently undergo curative resectional surgery. J Surg Oncol (1977) 9(4):347–52. doi: 10.1002/jso.2930090405 895156

[21] Ellis ER Mendenhall WM Rao PV McCarty PJ Parsons JT Stringer SP. Incisional or excisional neck-node biopsy before definitive radiotherapy, alone or followed by neck dissection. Head Neck (1991) 13(3):177–83. doi: 10.1002/hed.2880130303 2037468

[22] Holmes BJ Maleki Z Westra WH. The fidelity of p16 staining as a surrogate marker of human papillomavirus status in fine-needle aspirates and core biopsies of neck node metastases: Implications for HPV testing protocols. Acta Cytol (2015) 59(1):97–103. doi: 10.1159/000375148 25765380 PMC4406808

[23] Arsa L Siripoon T Trachu N Foyhirun S Pangpunyakulchai D Sanpapant S. Discrepancy in p16 expression in patients with HPV-associated head and neck squamous cell carcinoma in Thailand: clinical characteristics and survival outcomes. BMC Cancer (2021) 21(1):504. doi: 10.1186/s12885-021-08213-9 33957888 PMC8101232

[24] Beadle BM William WN Jr McLemore MS Sturgis EM Williams MD. p16 expression in cutaneous squamous carcinomas with neck metastases: a potential pitfall in identifying unknown primaries of the head and neck. Head Neck (2013) 35(11):1527–33. doi: 10.1002/hed.23188 23108906

[25] Chan JW Yeh I El-Sayed IH Algazi AP Glastonbury CM Ha PK. Ultraviolet light-related DNA damage mutation signature distinguishes cutaneous from mucosal or other origin for head and neck squamous cell carcinoma of unknown primary site. Head Neck (2019) 41(6):E82–5. doi: 10.1002/hed.25613 30633411

[26] Luo WJ Feng YF Guo R Tang LL Chen L Zhou GQ. Patterns of EBV-positive cervical lymph node involvement in head and neck cancer and implications for the management of nasopharyngeal carcinoma T0 classification. Oral Oncol (2019) 91:7–12. doi: 10.1016/j.oraloncology.2019.01.012 30926066

[27] Muraki AS Mancuso AA Harnsberger HR. Metastatic cervical adenopathy from tumors of unknown origin: the role of CT. Radiology (1984) 152:749–53. 10.1148/radiology.152.3.6463256 6463256

[28] Mendenhall WM Mancuso AA Parsons JT Stringer SP Cassisi NJ. Diagnostic evaluation of squamous cell carcinoma metastatic to cervical lymph nodes from an unknown head and neck primary site. Head Neck (1998) 20(8):739–44. doi: 10.1002/(sici)1097-0347(199812)20:8<739::aid-hed13>3.0.co;2-0 9790297

[29] Keller F Psychogios G Linke R Lell M Kuwert T Iro H. Carcinoma of unknown primary in the head and neck: comparison between positron emission tomography (PET) and PET/CT. Head Neck (2011) 33(11):1569–75. doi: 10.1002/hed.21635 21990221

[30] Majchrzak E Cholewiński W Golusiński W. Carcinoma of unknown primary in the head and neck: The evaluation of the effectiveness of (18)F-FDG-PET/CT, own experience. Rep Pract Oncol Radiother (2015) 20(5):393–7. doi: 10.1016/j.rpor.2015.07.002 PMC4597089 26549998

[31] Culverwell AD Scarsbrook AF Chowdhury FU. False-positive uptake on 2-[^18^F]-fluoro-2-deoxy-D-glucose (FDG) positron-emission tomography/computed tomography (PET/CT) in oncological imaging. Clin Radiol (2011) 66(4):366–82. doi: 10.1016/j.crad.2010.12.004 21356398

[32] Tanzler ED Amdur RJ Morris CG Werning JW Mendenhall WM. Challenging the need for random directed biopsies of the nasopharynx, pyriform sinus, and contralateral tonsil in the workup of unknown primary squamous cell carcinoma of the head and neck. Head Neck (2016) 38(4):578–81. doi: 10.1002/hed.23931 25488125

[33] Noor A Stepan L Kao SS Dharmawardana N Ooi EH Hodge JC. Reviewing indications for panendoscopy in the investigation of head and neck squamous cell carcinoma. J Laryngol Otol (2018) 132(10):901–5. doi: 10.1017/S0022215118001718 30289089

[34] Pattani KM Goodier M Lilien D Kupferman T Caldito G NCO. Utility of panendoscopy for the detection of unknown primary head and neck cancer in patients with a negative PET/CT scan. Ear Nose Throat J (2011) 90(8):E16–20. doi: 10.1177/014556131109000818 21853427

[35] Metzger K Horn D Pfeiffer T Moratin J Kansy K Ristow O. Is panendoscopy a necessary staging procedure in patients with lacking risk factors and oral squamous cell carcinoma? J Craniomaxillofac Surg (2019) 47(12):1968–72. doi: 10.1016/j.jcms.2019.11.009 31810847

[36] Durmus K Rangarajan SV Old MO Agrawal A Teknos TN Ozer E. Transoral robotic approach to carcinoma of unknown primary. Head Neck (2014) 36(6):848–52. doi: 10.1002/hed.23385 PMC4266274 23720223

[37] Hatten KM O’Malley BW Jr Bur AM Patel MR Rassekh CH Newman JG. Transoral robotic surgery-assisted endoscopy with primary site detection and treatment in occult mucosal primaries. JAMA Otolaryngol Head Neck Surg (2017) 143(3):267–73. doi: 10.1001/jamaoto.2016.3419 27930761

[38] Farooq S Khandavilli S Dretzke J Moore D Nankivell PC Sharma N. Transoral tongue base mucosectomy for the identification of the primary site in the work-up of cancers of unknown origin: Systematic review and meta-analysis. Oral Oncol (2019) 91:97–106. doi: 10.1016/j.oraloncology.2019.02.018 30926070

[39] Patel SA Magnuson JS Holsinger FC Karni RJ Richmon JD Gross ND. Robotic surgery for primary head and neck squamous cell carcinoma of unknown site. JAMA Otolaryngol Head Neck Surg (2013) 139(11):1203–11. doi: 10.1001/jamaoto.2013.5189 24136446

[40] Patel MR Ottenstein L Ryan M Farrell A Studer M Baddour HM. TORS elective lingual tonsillectomy has less acute morbidity than therapeutic base of tongue TORS. Oral Oncol (2021) 117:105294. doi: 10.1016/j.oraloncology.2021.105294 33878679 PMC8281337

[41] Kubik M Mandal R Albergotti W Duvvuri U Ferris RL Kim S. Effect of transcervical arterial ligation on the severity of postoperative hemorrhage after transoral robotic surgery. Head Neck (2017) 39(8):1510–5. doi: 10.1002/hed.24677 PMC5789773 28570011

[42] Gleysteen J Troob S Light T Brickman D Clayburgh D Andersen P. The impact of prophylactic external carotid artery ligation on postoperative bleeding after transoral robotic surgery (TORS) for oropharyngeal squamous cell carcinoma. Oral Oncol (2017) 70:1–6. doi: 10.1016/j.oraloncology.2017.04.014 28622885

[43] van Weert S Rijken JA Plantone F Bloemena E Vergeer MR Lissenberg-Witte BI. A systematic review on transoral robotic surgery (TORS) for carcinoma of unknown primary origin: Has tongue base mucosectomy become indispensable? Clin Otolaryngol (2020) 45(5):732–8. doi: 10.1111/coa.13565 PMC7496155 32369264

[44] Kubik MW Channir HI Rubek N Kim S Ferris RL von Buchwald C. TORS base-of-Tongue mucosectomy in human papilloma virus-negative carcinoma of unknown primary. Laryngoscope (2021) 131(1):78–81. doi: 10.1002/lary.28617 32239774

[45] Patel SA Parvathaneni A Parvathaneni U Houlton JJ Karni RJ Liao JJ. Post-operative therapy following transoral robotic surgery for unknown primary cancers of the head and neck. Oral Oncol (2017) 72:150–6. doi: 10.1016/j.oraloncology.2017.07.019 28797451

[46] Grau C Johansen LV Jakobsen J Geertsen P Anderson E Jensen BB. Cervical lymph node metastases from unknown primary tumours. results from a national survey by the Danish society for head and neck oncology. Radiother Oncol (2000) 55(2):121–9. doi: 10.1016/s0167-8140(00)00172-9 10799723

[47] Weir L Keane T Cummings B Goodman P O’Sullivan B Payne D. Radiation treatment of cervical lymph node metastases from an unknown primary: an analysis of outcome by treatment volume and other prognostic factors. Radiother Oncol (1995) 35(3):206–11. doi: 10.1016/0167-8140(95)01559-y 7480823

[48] Mourad WF Hu KS Shasha D Concert C Ishihara D Lin W. Initial experience with oropharynx-targeted radiation therapy for metastatic squamous cell carcinoma of unknown primary of the head and neck. Anticancer Res (2014) 34(1):243–8. 24403470

[49] Kennel T Garrel R Costes V Boisselier P Crampette L Favier V. Head and neck carcinoma of unknown primary. Eur Ann Otorhinolaryngol Head Neck Dis (2019) 136(3):185–92. doi: 10.1016/j.anorl.2019.04.002 31005456

[50] Sherry AD Pasalic D Gunn GB Fuller CD Phan J Rosenthal DI. Proton beam therapy for head and neck carcinoma of unknown primary: Toxicity and quality of life. Int J Part Ther (2021) 8(1):234–47. doi: 10.14338/IJPT-20-00034.1 PMC8270080 34285950

[51] Sher DJ Balboni TA Haddad RI Norris CM Jr Posner MR Wirth LJ. Efficacy and toxicity of chemoradiotherapy using intensity-modulated radiotherapy for unknown primary of head and neck. Int J Radiat Oncol Biol Phys (2011) 80(5):1405–11. doi: 10.1016/j.ijrobp.2010.04.029 21177045

[52] de Ridder M Klop M Hamming-Vrieze O de Boer J Jasperse B Smit L. Unknown primary head and neck squamous cell carcinoma in the era of fluorodeoxyglucose-positron emission tomography/CT and intensity-modulated radiotherapy. Head Neck (2017) 39(7):1382–91. doi: 10.1002/hed.24762 28370570

[53] Richards TM Bhide SA Miah AB Del Rosario L Bodla S Thway K. Total mucosal irradiation with intensity-modulated radiotherapy in patients with head and neck carcinoma of unknown primary: A pooled analysis of two prospective studies. Clin Oncol (R Coll Radiol) (2016) 28(9):e77–84. doi: 10.1016/j.clon.2016.04.035 27180092

[54] Grewal AS Rajasekaran K Cannady SB Chalian AA Ghiam AF Lin A. Pharyngeal-sparing radiation for head and neck carcinoma of unknown primary following TORS assisted work-up. Laryngoscope (2020) 130(3):691–7. doi: 10.1002/lary.28200 31411747

[55] Ang KK Harris J Wheeler R Weber R Rosenthal DI Nguyen-Tân PF. Human papillomavirus and survival of patients with oropharyngeal cancer. N Engl J Med (2010) 363(1):24–35. doi: 10.1056/NEJMoa0912217 20530316 PMC2943767

[56] Civantos FJ Vermorken JB Shah JP Rinaldo A Suárez C Kowalski LP. Metastatic squamous cell carcinoma to the cervical lymph nodes from an unknown primary cancer: Management in the HPV era. Front Oncol (2020) 10:593164. doi: 10.3389/fonc.2020.593164 33244460 PMC7685177

[57] LaVigne AW Margalit DN Rawal B Puzanov M Annino DJ Goguen LA. IMRT-based treatment of unknown primary malignancy of the head and neck: Outcomes and improved toxicity with decreased mucosal dose and larynx sparing. Head Neck (2019) 41(4):959–66. doi: 10.1002/hed.25531 30620435

[58] Ko HC Harari PM Sacotte RM Chen S Wieland AM Yu M. Prognostic implications of human papillomavirus status for patients with non-oropharyngeal head and neck squamous cell carcinomas. J Cancer Res Clin Oncol (2017) 143(11):2341–50. doi: 10.1007/s00432-017-2481-8 PMC7069668 28752235

[59] Chung CH Zhang Q Kong CS Harris J Fertig E Harari PM. p16 protein expression and human papillomavirus status as prognostic biomarkers of nonoropharyngeal head and neck squamous cell carcinoma. J Clin Oncol (2014) 32(35):3930–8. doi: 10.1200/JCO.2013.54.5228 PMC4251957 25267748

[60] Harris SL Thorne LB Seaman WT Hayes DN Couch ME Kimple RJ. Association of p16(INK4a) overexpression with improved outcomes in young patients with squamous cell cancers of the oral tongue. Head Neck (2011) 33(11):1622–7. doi: 10.1002/hed.21650 21990227

[61] Shaughnessy JN Farghaly H Wilson L Redman R Potts K Bumpous J. HPV: a factor in organ preservation for locally advanced larynx and hypopharynx cancer? Am J Otolaryngol (2014) 35(1):19–24. doi: 10.1016/j.amjoto.2013.08.006 24119488

[62] Sivars L Bersani C Grün N Ramqvist T Munck-Wikland E Von Buchwald C. Human papillomavirus is a favourable prognostic factor in cancer of unknown primary in the head and neck region and in hypopharyngeal cancer. Mol Clin Oncol (2016) 5(6):671–4. doi: 10.3892/mco.2016.1050 PMC5228397 28105346

[63] Lassen P Eriksen JG Krogdahl A Therkildsen MH Ulhoi BP Overgaard M. The influence of HPV-associated p16-expression on accelerated fractionated radiotherapy in head and neck cancer: evaluation of the randomised DAHANCA 6&7 trial. Radiother Oncol (2011) 100(1):49–55. doi: 10.1016/j.radonc.2011.02.010 21429609

[64] Cummings MA Ma SJ van der Sloot P Milano MT Singh DP Singh AK. Squamous cell carcinoma of the head and neck with unknown primary: trends and outcomes from a hospital-based registry. Ann Transl Med (2021) 9(4):284. doi: 10.21037/atm-20-4631 33708911 PMC7944267

[65] Madani I Vakaet L Bonte K Boterberg B De Neve W. Intensity-modulated radiotherapy for cervical lymph node metastases from unknown primary cancer. Int J Radiat Oncol Biol Phys (2008) 71(4):1158–66. doi: 10.1016/j.ijrobp.2007.11.059 18258383

[66] Ferris RL Flamand Y Weinstein GS Li S Quon H Mehra R. Phase II randomized trial of transoral surgery and low-dose intensity modulated radiation therapy in resectable p16+ locally advanced oropharynx cancer: An ECOG-ACRIN cancer research group trial (E3311). J Clin Oncol (2022) 40(2):138–49. doi: 10.1200/JCO.21.01752 PMC8718241 34699271

[67] Golusinski P Corry J Poorten VV Simo R Sjögren E Mäkitie A. De-escalation studies in HPV-positive oropharyngeal cancer: How should we proceed? Oral Oncol (2021) 123:105620. doi: 10.1016/j.oraloncology.2021.105620 34798575

[68] Cheraghlou S Torabi SJ Husain ZA Otremba MD Osborn HA Mehra S. HPV status in unknown primary head and neck cancer: Prognosis and treatment outcomes. Laryngoscope (2019) 129(3):684–91. doi: 10.1002/lary.27475 30151832

[69] Bollig CA Morris B Stubbs VC. Transoral robotic surgery with neck dissection versus nonsurgical treatment in stage I and II human papillomavirus-negative oropharyngeal cancer. Head Neck (2022) 44(7):1545–53. doi: 10.1002/hed.27045 PMC9324989 35365915

